# Simplified Acute Physiology Score II as Predictor of Mortality in Intensive Care Units: A Decision Curve Analysis

**DOI:** 10.1371/journal.pone.0164828

**Published:** 2016-10-14

**Authors:** Jérôme Allyn, Cyril Ferdynus, Michel Bohrer, Cécile Dalban, Dorothée Valance, Nicolas Allou

**Affiliations:** 1 Intensive care unit, Saint-Denis University Hospital, Saint-Denis, Reunion Island, France; 2 Unité de Soutien Méthodologique, Saint-Denis University Hospital, Saint-Denis, Reunion Island, France; 3 INSERM, CIC 1410, Saint-Pierre, Reunion Island, France; 4 Department of Medical Information, Saint-Denis University Hospital, Saint-Denis, Reunion Island, France; Azienda Ospedaliero Universitaria Careggi, ITALY

## Abstract

**Background:**

End-of-life decision-making in Intensive care Units (ICUs) is difficult. The main problems encountered are the lack of a reliable prediction score for death and the fact that the opinion of patients is rarely taken into consideration. The Decision Curve Analysis (DCA) is a recent method developed to evaluate the prediction models and which takes into account the wishes of patients (or surrogates) to expose themselves to the risk of obtaining a false result. Our objective was to evaluate the clinical usefulness, with DCA, of the Simplified Acute Physiology Score II (SAPS II) to predict ICU mortality.

**Methods:**

We conducted a retrospective cohort study from January 2011 to September 2015, in a medical-surgical 23-bed ICU at University Hospital. Performances of the SAPS II, a modified SAPS II (without AGE), and age to predict ICU mortality, were measured by a Receiver Operating Characteristic (ROC) analysis and DCA.

**Results:**

Among the 4.370 patients admitted, 23.3% died in the ICU. Mean (standard deviation) age was 56.8 (16.7) years, and median (first-third quartile) SAPS II was 48 (34–65). Areas under ROC curves were 0.828 (0.813–0.843) for SAPS II, 0.814 (0.798–0.829) for modified SAPS II and of 0.627 (0.608–0.646) for age. DCA showed a net benefit whatever the probability threshold, especially under 0.5.

**Conclusion:**

DCA shows the benefits of the SAPS II to predict ICU mortality, especially when the probability threshold is low. Complementary studies are needed to define the exact role that the SAPS II can play in end-of-life decision-making in ICUs.

## Introduction

The management of patients in Intensive Care Units (ICUs) often requires reflection on a possible decision to forego hopeless life-sustaining treatment. [1,2]

A recent French multicenter study reported that 14% of admitted patients and 52% of patients dying in an ICU had their treatment withheld or withdrawn [3]. Ideally, it is the patient who makes the decision to withhold or withdraw life-sustaining medical treatment, whether he/she is fit or has previously expressed wishes about care. If the patient cannot make this decision, a surrogate may interfere; however, in all cases, physicians must provide clear and accurate information.

The two main difficulties encountered in end-of-life decision-making are the lack of a reliable prediction score for death and the fact that the opinion of patients is too rarely taken into consideration [4]. Several studies have shown great variability in end-of-life decisions across world regions and countries [5–8]. Age and the SAPS II are significantly related to the decision to withhold or withdraw life-sustaining medical treatment [3,9].

The Simplified Acute Physiology Score II (SAPS II) was developed and validated in a cohort of more than 13,000 patients in 13 Intensive Care Units. The SAPS II helps to predict in-hospital mortality thanks to the collection of 17 variables at ICU admission, without considering the reason for admission [10]. The goal of the authors was to establish a criterion for evaluating the performance of ICUs. Since the original study by Le Gall et *al*., this score has been the subject of many studies that are of relevance to specific groups of patients (severe trauma, infection, circulatory assisted patients, cardiac surgery, etc.), or else to mortality prediction (for example, in the case of occurrence of nosocomial infection) [11–17]. These studies typically use a methodology based on multi-varied analysis using logistic regression and Receiver Operating Characteristic (ROC) curves. Like all scores, the SAPS II is not used in routine practice and especially not to make end-of-life decisions in ICUs.

The Decision Curve Analysis (DCA) was developed to evaluate and compare the diagnostic and prediction models by integrating the clinical consequences of false positive and negative results [18]. Among many other advantages, this method takes into account the wishes of patients (or surrogates) to expose themselves to the risk of obtaining a false negative or a false positive. The results are presented as a graph with the selected probability threshold plotted on the abscissa and the benefit of the evaluated model on the ordinate.

This evaluation method may be of considerable help for making decisions on medical treatment with heavy side effects, and several studies have been conducted as part of the diagnosis of cancer [19–22]. To our knowledge, only one study based on this method was conducted outside the context of cancer, namely in the context of sepsis [23].

We hypothesize that the SAPS II may be of considerable help for end-of-life decision-making. Our main objective was to evaluate the clinical usefulness of the SAPS II, the modified SAPS II (without the “age” variable), and age to predict ICU mortality. We conducted a DCA to (i) integrate the risk of unnecessary resuscitation (futile aggressive treatment) as well as the risk of unhappy end-of-life decision-making (avoidable death), and to (ii) integrate the wishes of patients (or surrogates). Our secondary objective was to compare the clinical usefulness of the SAPS II to predict ICU mortality depending of the reason for admission. To the best of our knowledge, the SAPS II has never been evaluated via a DCA before.

## Methods

### Study population and data collection

We conducted a retrospective observational study in a 23-bed adult medical-surgical ICU at a French university hospital between January 2011 and September 2015. We retrospectively analyzed data from a healthcare database for all patients admitted to the ICU during this period (S1 Table). Patient identification and data collection were performed by the hospital’s information department. In the ICU, the SAPS II was calculated for each admission one day after admission, based on 17 variables, as described in the article of Le Gall *et al*. [10]. The medical team in the ICU does not currently use the SAPS II in medical discussions.

Data on age, sex, reason for admission (ICD10 codes), SAPS II, modified SAPS II (SAPS II without the AGE variable) and evolution in ICU were collected. The primary outcome was the rate of ICU mortality. The patient records were de-identified and analyzed anonymously.

This study was approved by the Institutional Review Board (reference R15013). Reporting of this study complies with the Strengthening the Reporting of Observational studies in Epidemiology recommendations statement for reporting. [24]

### Statistical analysis

Qualitative variables were expressed as frequency, percentages, and 95% confidence intervals (95% CI). Quantitative variables were expressed as mean and standard deviation (SD) or median and first-third quartiles. Comparisons of percentages were performed by Chi-square test or by Fisher’s exact test, as appropriate. Comparisons of means were performed by Student t-test or by Mann and Whitney test, as appropriate.

Diagnostic performance of SAPS II, modified SAPS II and Age was assessed by sensitivity, specificity, positive and negative predictive values, and diagnostic accuracy. Receiver operating characteristic (ROC) curves, areas under curves (AUC) and their 95% Confidence Intervals (95% CI) were estimated for SAPS II, modified SAPS II and Age. Comparisons between ROC Curves were performed using the method described by DeLong *et al*. [25].

Finally, we analyzed the net benefit of SAPS II, modified SAPS II and Age for predicting ICU mortality. For these three methods, we calculated the net benefit using Decision curve analysis (DCA), as described by Vickers *et al*.: it consists in the subtraction of the proportion of all patients who are false-positive from the proportion who are true-positive, weighting by the relative harm of a false-positive and a false-negative result. [18]

$$Net\;benefit=\frac{True\;Positives}{n}-\left(\frac{p_{t}}{1-p_{t}}\right)\frac{False\;Positives}{n}$$

where n is the total number of patients included in the study and Pt is the probability threshold [18,26,27]. For example, a net benefit of 0.03 for a Pt of 50% can be interpreted as: « using this predictive model, 3 patients per 100 patients who will die in ICU will be placed under palliative care without increasing of patient under palliative care undue». The decision curve is constructed by varying Pt and plotting net benefit on the y vertical axis against Pt on the x horizontal axis.

DCA was performed using the SAS macro provided by Vickers *et al*. DCA was performed for all patients included in the study and, in the sensitivity analysis, for patients stratified according to major reason for ICU admission: cardiogenic shock (R570), hypovolemic shock (R571), septic shock (R572), coma (R40.x), and respiratory distress syndrome (J80, J96.x). All statistical tests were performed at the two-tailed level of significance at 5%. All analyses were performed using SAS 9.4 (SAS Institute, Inc, Cary, NC, USA).

## Results

### Characteristics of patients at ICU admission

During the 4 years and 9 months study period, 4,370 patients were admitted to the ICU (Table 1). Among them, 1,018 (23.3%; 95%CI: 22.0–24.5) died in the ICU in 7.2 (10.4) days. The intrahospital mortality was not available in the database. The demographics characteristics and the reason for admission of the 4,370 patients are presented in Table 1. The mean age was 56.8 (16.7), and the median SAPS II at admission was 48.0 (34–65). The predicted in-hospital mortality rate was 41.5%, based on the original formula of Le Gall *et al*. [10].

**Table 1:** Characteristics of patients at admission and evolution in ICU.

	Total (n = 4370)	Alive (n = 3352)	Dead (n = 1018)	*p-value*
Age (years) ^†^	56.8 (16.7)	55.1 (17.0)	62.6 (14.2)	< 0.0001
Sex (male), n (%)	2691 (61.6%)	2057 (61.4%)	634 (62.3%)	0.60
SAPS II at admission^§^	48 (34–65)	43 (31–57)	72 (57–89)	< 0.0001
Modified SAPS II at admission^(^^*^^)^^§^	39 (25–55)	34 (23–47)	61 (45–78)	< 0.0001
Reason for ICU admission, n (%)				< 0.0001
Infectious diseases	290 (6.6%)	222 (6.6%)	68 (6.7%)	
Diseases of the nervous system	201 (4.6%)	183 (5.5%)	18 (1.8%)	
Cardiovascular diseases	609 (13.9%)	357 (10.6%)	252 (24.7%)	
Diseases of the respiratory system	1077 (24.6%)	897 (26.8%)	180 (17.7%)	
Diseases of the digestive system	136 (3.1%)	116 (3.5%)	20 (1.9%)	
Coma	337 (7.7%)	284 (8.5%)	53 (5.2%)	
Shocks	1148 (26.3%)	791 (23.6%)	357 (35.1%)	
Other diseases	572 (13.1%)	502 (14.9%)	70 (6.9%)	
Length of stay in ICU (days) ^†^	7.3 (9.7)	7.4 (9.5)	7.2 (10.4)	0.69

(*) modified SAPS II at admission is defined by calculating SAPS II without the AGE variable.

† data are expressed as mean and standard deviation.

§ data are expressed as median and first-third quartiles.

### ROC Analysis

**Fig 1:**
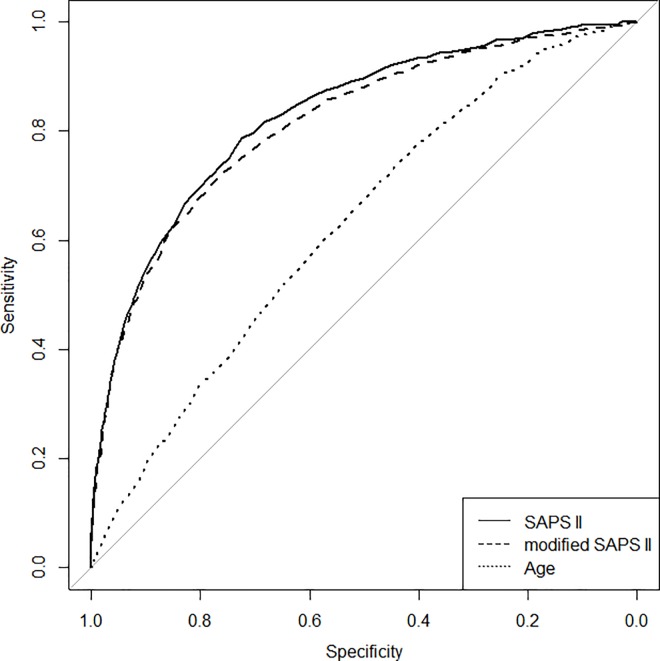
Receiver operating characteristic curves showing the performances of SAPS II, modified SAPS II and age to predict ICU mortality. Areas under curves (95% CI) are 0.828 (0.813–0.843), 0.814 (0.798–0.829), and 0.627 (0.608–0.646).

Fig 1 presents the ROC curves that show the performances of SAPS II, modified SAPS II, and age to predict ICU mortality for all patients included in the study. Areas under curves (95% CI) were respectively 0.828 (0.813–0.843), 0.814 (0.798–0.829), and 0.627 (0.608–0.646). The area under the ROC curve (AUC) for SAPS II was significantly different from the AUCs for modified SAPS II (p < 0.0001) and age (p < 0.0001). The diagnostic performance of SAPS II in predicting ICU mortality is presented in Table 2.

**Table 2:** Diagnostic performance of SAPS II in predicting ICU mortality.

Values of SAPS II	Sensitivity	Specificity	Accuracy	Negative predictive value	Positive predictive value
**> 10**	100%	1.0%	24.1%	23.5%	100%
**> 20**	99.4%	9.1%	30.2%	24.9%	98.1%
**> 30**	96.8%	23.8%	40.8%	27.8%	96.0%
**> 40**	92.1%	44.7%	55.8%	33.6%	94.9%
**> 50**	83.2%	64.9%	69.1%	41.8%	92.7%
**> 60**	69.7%	80.1%	77.7%	51.6%	89.7%
**> 70**	53.3%	90.7%	82.0%	63.6%	86.5%
**> 80**	37.0%	95.9%	82.2%	73.5%	83.4%
**> 90**	23.0%	98.2%	80.7%	79.9%	80.8%
**> 100**	10.5%	99.7%	78.9%	91.5%	78.6%

### Decision Curve Analysis

**Fig 2:**
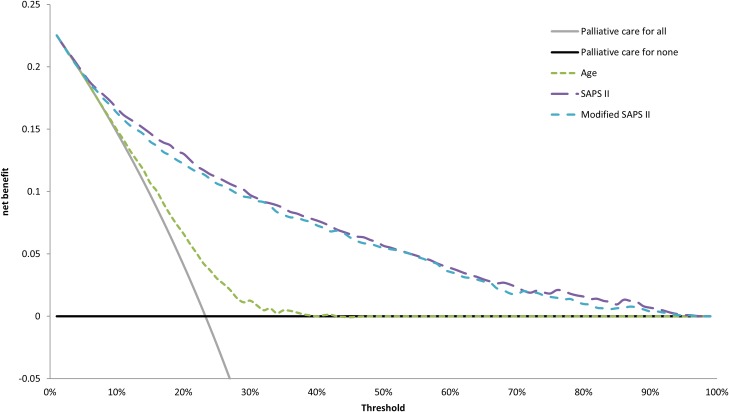
Decision curves showing the clinical usefulness of SAPS II, modified SAPS II and age to predict ICU mortality. Solid black line represents the net benefit of applying palliative care for no patients, assuming that all patients would be alive. Solid gray line represents the net benefit of applying palliative care for all patients, assuming that all would die. Long dashed line, medium dash line and short dash line represent the net benefit of applying palliative care to patients according to SAPS II, modified SAPS II and age, respectively.

Fig 2 presents the decision curves that show the clinical usefulness of SAPS II, modified SAPS II, and age to predict ICU mortality in the total population of patients. The results are presented as a graph with the selected probability threshold (*i*.*e*., the degree of certitude of ICU mortality over which the patient's decision is to be placed in palliative care) plotted on the abscissa and the benefit of the evaluated model on the ordinate [18,28]. The curves of the 3 models do not intersect, and the curves of the SAPS II and the modified SAPS II remain very close regardless of the selected threshold. The SAPS II model provides a net benefit regardless of the selected threshold, and the net benefit is greater than 0.1 when the threshold is smaller than 0.3. The model based on age is very inefficient, with no benefit when the threshold is greater than 40%.

Observed mortality, predicted mortality and net benefit of SAPS II are presented in Table 3.

**Table 3:** Predicted ICU mortality and net benefit of SAPS II.

Value of SAPS II	Predicted ICU mortality (%)	95% CI of predicted ICU mortality	Net benefit
**10**	1.4%	1.1%– 1.8%	0.225
**20**	2.6%	2.2% - 3.2%	0.209
**30**	4.9%	4.2% - 5.7%	0.192
**40**	8.9%	7.9% - 10.0%	0.158
**50**	15.6%	14.3% - 16.9%	0.142
**60**	25.9%	24.3% - 27.5%	0.109
**70**	39.8%	37.7% - 42.0%	0.077
**80**	55.6%	52.8% - 58.4%	0.047
**90**	70.4%	67.2% - 73.3%	0.023
**100**	81.8%	78.9% - 84.4%	0.014

Predicted mortality was calculated by introducing SAPS II as continuous covariate in a univariate logistic regression.

### Sub-group analysis

**Fig 3:**
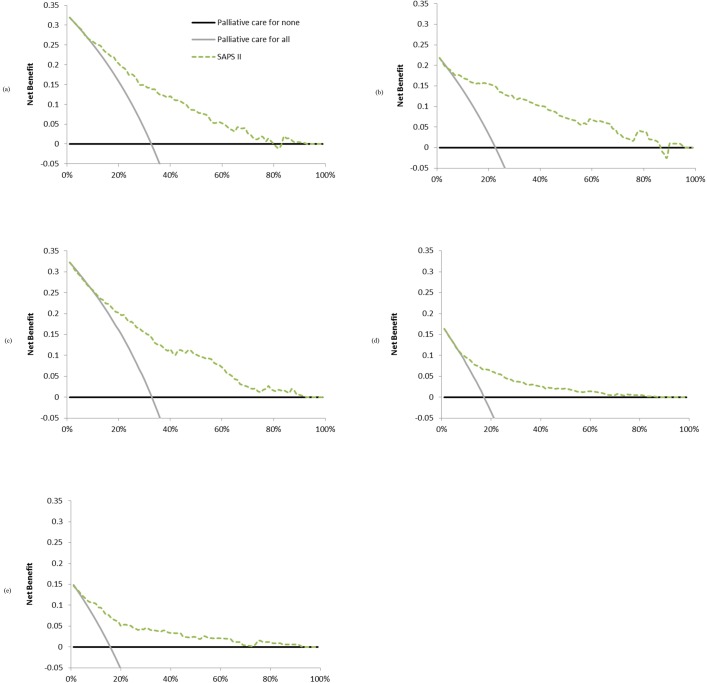
Decision curves according to reason for ICU admission to predict ICU mortality. Decision curves showing the clinical usefulness of SAPS II to predict ICU mortality according to major reason for ICU admission: (a) cardiogenic shock, (b) hypovolemic shock, (c) septic shock, (d) coma and (e) respiratory distress syndrome. Solid black line represents the net benefit of applying palliative care for no patients, assuming that all patients would be alive. Solid gray line represents the net benefit of applying palliative care for all patients, assuming that all would die. Dashed line represents the net benefit of applying palliative care to patients according to SAPS II.

Complementary analyses according to reason for ICU admission are shown in Fig 3.

## Discussion

To the best of our knowledge, our study is the first to use a DCA in the context of end-of-life decision-making in ICUs, even though this technique was developed precisely to help make difficult decisions—*i*.*e*., to deal with situations in which false results, whether negative or positive, can have serious consequences.

Indeed, end-of-life decisions are very difficult to make and are also quite random [5–8]. Moreover, no score has been developed to help with end-of-life decision-making [29]. It is obvious that a prognostic score can play a role in this area. Ideally, this score should have a sensitivity and specificity of 100%. Age and the SAPS II are significantly related to the decision to withhold or withdraw life-sustaining medical treatment [3,9]. It is this observation that prompted us to evaluate the ability of the SAPS II, the modified SAPS II (without the “age” variable), and age to predict ICU mortality.

The ROC analysis does not warrant using the SAPS II as an aid to end-of-life decision-making in ICUs. Here we show, on the one hand, that the DCA can be used for specific problems of intensive care medicine, and, on the other hand, that the DCA can bring new elements to end-of-life decision-making in ICUs. This method of analysis is particularly useful in this area because it is difficult to conduct studies with a more robust design, and this for ethical reasons

We studied a large cohort of patients with a median SAPS II of 48. Based on the original formula developed by Le Gall *et al*., this SAPS II predicted a mortality rate of 41.5%, which is far superior to the mortality rate of 23.3% observed in the ICU [10]. By comparison, the most recent French study on this subject reported an equivalent ICU mortality rate (ICU mortality rate of 20% for patients aged over 64, though with a lower SAPS II of 40) [3].

We showed, based on a large cohort that the SAPS II may be of considerable help for end-of-life decision-making in ICUs. This score provides a net benefit regardless of the selected threshold, and especially when the latter is lower than 0.5, *i*.*e*., when the degree of certitude of ICU mortality over which the patient's decision is to be placed in palliative care is intermediate or low. For high degrees of certitude, the SAPS II is less informative; in such cases, it might prove useful to use an alternative score, for instance the SOFA score [30].

This, then, is a new potential application for the SAPS II, which was originally developed to predict in-hospital mortality rates and to assess the quality of care. However, the SAPS II does not integrate the status of patients at ICU discharge, which would have been desirable for this score to help with end-of-life decision-making. It is obvious that the score alone will never replace human decision-making in this type of situation.

It may be that the SAPS II is more or less effective depending on the subgroup examined. Indeed, the subgroup analysis we conducted according to reason for admission produced quite different curves. For example, the SAPS II seems to be less useful for patients admitted for coma or respiratory distress than it is for patients admitted for any kind of shock.

Our study has several limitations. First, because of the retrospective nature of the study, bias may exist, and the reproducibility of the SAPS II cannot be validated. This point has already been discussed, namely in a study which reported an excellent intraclass correlation coefficient (95% CI) at 0.84 (0.74, 0.91) [31]. Moreover, we cannot provide neither the proportion of withhold or withdraw life-sustaining medical treatment, nor the intra-hospital mortality. Second, we could not evaluate other scores, which would have been useful for decision-making on the right section of the graph; perhaps it is in this part that the need is greatest. Scores calculated on the time of the decision making and not on admission in ICU could also be evaluate. We cannot exclude a bias related to the fact that patient management was modified on the basis of the SAPS II calculation after one day of hospitalization, even if this is not the usual practice. Finally, to our knowledge, no test has been developed to compare the curves.

## Conclusion

In conclusion, the SAPS II predicts ICU mortality and the DCA shows the benefits of this model, especially when the probability threshold is low. Complementary studies are needed to define the exact role that the SAPS II can play in end-of-life decision-making in ICUs.

## Supporting Information

S1 Table Dataset. Patient’s data (age, sex, death in Intensive Care Unit, length of stay in Intensive Care Unit, reason of admission).
(XLS)
